# Defining genetic architecture of the populations in the Indian subcontinent: Impact of human leukocyte antigen diversity studies

**DOI:** 10.4103/0971-6866.73394

**Published:** 2010

**Authors:** N. K. Mehra

**Affiliations:** Department of Transplant Immunology and Immunogenetics, All India Institute of Medical Sciences, New Delhi - 110 029, India

The post-genomics era has ushered in a renewed interest in genomic diversity studies particularly with regard to definition of polymorphisms and their functional significance. An understanding of the functionality of these large sequences and their variability between populations and individuals appears to be the next logical step to provide meaning to the voluminous human genome sequences. Population-based description of polymorphisms, especially in functionally active gene complexes, such as the major histocompatibility complex (MHC), has gained immense importance in this era of ‘functional genomics’. Because of the large degree of polymorphism and extensive linkage of its various loci, gene complex within the human leukocyte antigen (HLA) region, which is the human equivalent of MHC, serves as a good mini genome model for anthropological investigations of genetic relatedness and migrations of different world populations. Three outstanding features of the human MHC, namely high polymorphism, tight linkage among the loci, and the non-random association of its various alleles make the system of particular interest from the perspective of population genetics.

The Indian subcontinent is a land of enormous cultural, geographical, and linguistic diversity, and harbors appreciably greater genetic diversity than any other comparable global region after Africa. The society comprises various tribal groups, ranked caste and other population groups that are largely endogamous. Why and how this genetic diversity arose in India is not clear? The “Out of Africa” migration model of modern humans suggests that a small population, most probably from East Africa expanded throughout Africa around 100 kya. This was followed by a second expansion between 60 and 40 kya into Asia and from there to other continents.[1] Currently, it is widely accepted that the modern human evolved in Africa, migrated out of the continent and one of these major waves came to India and this served as a major platform for the dispersal of modern human to other geographical regions, including south-east Asia. Adding to the complexity of genetic diversity in the Indian population, several successive waves of migration and invasions from the Middle East, Central Asia as well as Mongolia have contributed to the available gene pool. From fourth century bc onwards, India particularly the northern region experienced several waves of immigrants for almost 200 years. History testifies Greeks, Pathans, Sakas and Pahlavas including the Kushans were the first to come after the Indo-Aryans civilization entered. On the west coast, Jews and Parsis came after fleeing from their own homelands. Similarly Arabian Muslims, Persian Muslims, Turks, and Afghans whose total number was very large came to India from time to time. The Muslim immigration into India began even before the Arab invasion of Sind, quite early in the eighth century AD,[2] and ended with the establishment of the Mughal Empire in the 16^th^ century. This was the last major movement, which produced perceptible changes in the composition and culture of the indigenous population. The Muslims did not accept the Hindu religion and this led to large-scale conversions. The Europeans, Portuguese, Dutch, French, and British started their intrusions from the 15^th^ century ad onwards, and the Indo-European community grew from these infusions. The Mongolians entered the eastern part of India from Southeast Asia. The Indian subcontinent therefore became the melting pot of the various racial groups who largely halted here and intermingled with the local population.

As a result of evolutionary history, endogamy, and consanguinity, populations of the Indian subcontinent demonstrate high genetic differentiation and extensive sub-structuring.[3] A recent study involving 25 well-defined ethnic groups in India along with Europeans and Chinese populations, concluded that the ancestry is unique to India.[4] The authors suggested that Indian groups have inherited different proportions of ancestry from the ancestral North Indians and the ancestral South Indians. Hence, consistent with social history, northern regions show closer affinities with Indo-European speaking populations of central Asia as compared to those inhabiting southern regions. Indeed the southern Indian population may have been derived from early colonizers arriving from Africa along the southern exit route.

HLA-based studies carried out in the Indian population by various groups.[5] have highlighted unique and extensive MHC diversity of the Indian population. For example, the HLA-A2 and A33 allele families in the Indian population show greater diversity with the existence of several ‘unique’ alleles. The best example is the presence of A*0211, an allele that occurs only in the Indian populations with almost complete absence in the Caucasoid and Oriental groups.[6] Similarly A*3303 that primarily occurs in the Mongoloid populations has been found with an appreciably high frequency in North Indians. Great diversity has also been observed in HLA class II allelic groups in this population. Amongst the haplotypes, the three most common HLA haplotypes that occur in North India are HLA-A33-B44-DR7, HLA-A33-B58-DR3, and HLA-A2-B50-DR3. Of these, HLA-A33-B44-DR7 is also observed in most other Asian countries like Malaysia, Thailand, South Korea, Bangladesh, Pakistan, China, but not reported in rest of the world. Similarly, HLA-A33-B58-DR3 is one of the commonest haplotypes in the Chinese population, which occurs with significantly high frequency in the Indian subcontinent suggesting racial admixture from the East. On the other hand, haplotype HLA-A2-B50-DR3 is observed exclusively in India particularly in patients with type 1 diabetes and is therefore ‘unique’ to this population. Since HLA genes play an important role in immune surveillance, it includes various infectious (HIV and TB) and autoimmune disease, associated or protective, genes and haplotypes. Nevertheless these associations reveal population-specific variations. For example, the most commonly encountered haplotype in the Europeans and North American Caucasoids that shows a strong association with autoimmune diseases is HLA-A1-B8-DR3, often referred to as the ancestral haplotype 8.1 (AH 8.1). The Indian population, on the other hand, does not follow the Caucasian trend, and AH 8.1 occurs either with a very low frequency or is absent in the subcontinent. Instead the haplotype HLA-A26-B8-DR3 designated as AH8.2[7] is the most frequently occurring haplotype in the Indian subcontinent, and this shows strong association with several autoimmune diseases just as AH 8.1 does in the Caucasians.[8] Major lessons drawn from these studies are summarized as under:

- Most populations in the Indian subcontinent follows the Caucasoid pattern of HLA allelic distribution with variable admixture of genes from the Mongoloid and Aryan races;
- The observed extreme diversity in the HLA class I and class II region genes in these populations is due primarily to the existence of several ‘novel alleles’ and ‘unique haplotypes’ that could have arisen as a consequence of the founder effect, racial admixture, and geophysical or socioeconomic barriers contributing to the genetic drift;
- There is compelling evidence to suggest that selection pressure could have directed the generation of such novel alleles and unique haplotypes.
- The high level of variance in the repertoire of peptide presenting MHC molecules implies altered antigen presentation, immune responses, and HLA genetic profiles in relation to disease susceptibility and resistance.
- The extreme degree of polymorphism may pose limitations for the development of potentially global vaccines and may require development of novel vaccination protocols for effective therapeutic use in extremely diverse populations like those in the subcontinent.
- The existence of large variety of alleles and haplotypes, both unique and representative of other ethnic groups in the Indian subcontinent poses additional challenges in the transplantation context, particularly with regard to the hematopoietic stem cell transplantation.

In view of the importance of HLA in defining genetic architecture of the human population, understanding the demographic history and population kinship along with unique constitution of MHC in ethnically well-defined populations has important implications on its immunobiology. This requires the use of advanced molecular technologies determining HLA diversity at high-resolution level, use of appropriate statistical methods for measuring the selection pressure (balancing or directional, etc.), maximum likelihood analysis, Ewans–Watterson homozygosity, strict selection of sampling algorithm to avoid sampling errors for better representation of a population, and critical evaluation of the data in relation to other populations. Owing to its inherent genetic diversity and ‘unique’ MHC repertoire, data generated in the Indian subcontinent on diverse population groups could become an invaluable genomic resource for assessing the implications of new polymorphisms in the generation of immune response, defining susceptibility/resistance factors in diseases, survival in varying environmental conditions, design of universal drugs or vaccines and finally to generate an insight into the extent and comparative effect of different evolutionary forces like founder effect, isolation, divergence, genetic admixture, and mutations, as well as environmental selection on the generation and maintenance of polymorphism.
